# Predictive value of left atrial strain analysis in adverse clinical events in patients with hypertrophic cardiomyopathy: a CMR study

**DOI:** 10.1186/s12872-023-03069-2

**Published:** 2023-01-23

**Authors:** Di Tian, JingYu Zhang, YiFan He, ZiQi Xiong, Min Zhao, Shuai Hu, QingWei Song, ZhiYong Li

**Affiliations:** 1 Department of Radiology, The First Affiliated Hospital of Dalian Medical University, Zhongshan Road No. 222, Xigang District, Dalian, 116011 China; 2 Pharmaceutical Diagnostics, GE Healthcare, Beijing, China

**Keywords:** Hypertrophic cardiomyopathy, Cardiac magnetic resonance, Left atrium, Strain, Risk factors

## Abstract

**Background:**

A subset of patients with hypertrophic cardiomyopathy (HCM) will experience adverse clinical events such as heart failure (HF), cardiovascular death, and new-onset atrial fibrillation (AF). Current risk stratification methods are imperfect and limit the identification of patients at high risk for HCM. This study aimed to evaluate the role of cardiac magnetic resonance (CMR)-derived left atrial strain parameters in the occurrence of adverse clinical events in patients with HCM.

**Methods:**

Left atrial (LA) structural, functional, and strain parameters were evaluated in 99 patients with HCM and compared with 89 age-, sex-, and BMI-matched control subjects. LA strain parameters were derived from CMR two- and four-chamber cine images by a semiautomatic method. LA strain parameters include global longitudinal strain (GLS) and global circumferential strain (GCS). The LA GLS includes reservoir strain (GLS reservoir), conduit strain (GLS conduit), and booster strain (GLS booster). Three LA GLS strain rate (SR) parameters were derived: SR reservoir, SR conduit, and SR booster. The primary endpoint was set as a composite of adverse clinical events, including SCD, new-onset or worsening to hospitalized HF, new-onset AF, thromboembolic events, and fatal ventricular arrhythmias.

**Results:**

LA GLS, GLS SR and GCS were impaired in HCM patients compared to control subjects (all *p* < 0.001). After a mean follow-up of 37.94 ± 23.69 months, 18 HCM patients reached the primary endpoint. LA GLS, GLS SR, and GCS were significantly lower in HCM patients with adverse clinical events than in those without adverse clinical events (all *p* < 0.05). In stepwise multiple Cox regression analysis, LV SV, LA diameter, pre-contraction LAV (LAV pre-ac), passive LA ejection fraction (EF), and LA GLS booster were all independent determinants of adverse clinical events. LA GLS booster ≤ 8.9% was the strongest determinant (HR = 8.9 [95%CI (1.951, 40.933)], *p* = 0.005). Finally, LA GLS booster provided predictive adverse clinical events value (AUC = 0.86 [95%CI 0.77–0.98]) that exceeded traditional outcome predictors.

**Conclusion:**

LA strain assessment, a measure of LA function, provides additional predictive information for established predictors of HCM patients. LA GLS booster was independently associated with adverse clinical events in patients with HCM.

## Introduction

Hypertrophic cardiomyopathy (HCM) is an autosomal dominant disorder with an incidence of approximately 1:500–1:200 worldwide [1]. Patients with HCM have a higher incidence of cardiovascular disease and mortality than the general population [2, 3]. However, identifying patients with high-risk HCM remains challenging [4]. The 2014 European sudden cardiac death (SCD) risk prediction model for HCM includes left atrium (LA) diameter as one of its indicators [5]. Established studies have shown that LA size and LA volume are significantly associated with adverse clinical outcomes in patients with HCM, including atrial fibrillation (AF), thromboembolic events, heart failure (HF), and death [6–9]. Nevertheless, these parameters alone are insufficient to describe how complicated the LA function is. Recently, LA strain is a promising parameter for quantifying LA phase function and may better reflect LA pathophysiology [10, 11], and provide additional prognostic value for HCM [12–14].

LA strain can reflect the function of the three phases of LA, including the reservoir phase, the conduit phase, and the pumping phase. Several studies have found that CMR-FT assessment of LA strain has good feasibility and reliability in normal populations [15–17]. In addition, Yang et al. [18] recently demonstrated that CMR-FT can also accurately and reproducibly assess LA strain with HCM.

LA strain is increasingly recognized as having an increasingly important role in determining the prognosis and risk stratification of cardiac patients, such as acute myocardial infarction [19], HF [20], dilated cardiomyopathy [21], and HCM [22]. There are only three studies assessing adverse clinical outcomes in patients with HCM based on the CMR-FT using the left atrial strain approach. Hinojar et al. [13] showed that the LA strain may be a new predictor of adverse cardiac events in patients with HCM. However, the three phases of LA strain were not subdivided in this study. Zhou et al. [23] used standard CMR-FT two-chamber, three-chamber, and four-chamber views to assess adverse outcomes in HCM patients. It was shown that LA reservoir strain and booster strain were associated with adverse outcomes. Based on the CMR-FT two-chamber and four-chamber views, Yang et al. [14] analyzed the prognosis of these patients with HCM through the fast semi-automated left atrial strain. They found that these patients’ LA reservoir strain and conduit strain are correlated with adverse clinical outcomes. However, the results of the available studies are not entirely consistent. We measured LA strain in standard CMR-FT two- and four-chamber views, which is the method used in most studies. This study further investigates the predictive value of LA strain on adverse clinical outcomes in patients with HCM. In addition, the predictive performance of different parameters for predicting adverse clinical events in HCM patients at the 3-year time point was assessed using the receiver operating characteristic (ROC) curve, and subsequently a Cox nomogram was constructed that can be used more intuitively and easily to guide clinical decision making.

## Materials and methods

### Study population

According to ESC diagnostic criteria [5], patients with genetically diagnosed or familial HCM who had wall thickness ≥ 13 mm, or non-familial HCM patients with wall thickness ≥ 15 mm but no other cause of hypertrophy was found were included in this study. Patients with HCM were included in this study regardless of the presence or absence of systolic dysfunction. Participants were enrolled from May 2012 to September 2021. Exclusion criteria included known causes of cardiac hypertrophy; previous, or current atrial fibrillation; known contraindications to CMR imaging (e.g., severe claustrophobia, pacemakers in non-MRI conditions).In addition, 89 control subjects matched for age, sex, and BMI were selected. These controls were community-derived, had no known cardiovascular disease or family history of heart disease and had a normal electrocardiogram.

This study was approved by our institution and written informed consent was waived due to its retrospective nature (Approval number: YJ-KS-KY-2022-238).

### CMR scan protocol

Images were acquired using two 3.0-T MRI scanners, including GE Signa HDxt MR (Waukesha, WI, USA) and Philips Ingenia (Philips Healthcare, Cleveland, Ohio, USA). An 8- and 16-channel coil was used, respectively. All scans are performed using cardiac gating and respiratory gating technology. Patients were also trained to breathe before scanning. Conventional cine imaging is acquiring MR signals in multiple heartbeats.Left ventricular (LV) long-axis two-chamber and four-chamber heart cine images were acquired with the following sequence parameters for the Signa HDx scanner: TR 3.6 ms; TE 1.6 ms; FOV 350 mm × 350 mm; flip angle 50°; matrix 192 × 224; layer thickness 10 mm; layer spacing 0 mm. Sequence parameters for the Philips Ingenia scanner were: TR 2.6–3.0 ms; TE 1.31–1.51 ms; FOV 380 mm × 380 mm; flip angle 50°; matrix 192 × 155; layer thickness 8 mm; layer spacing 0 mm. Cine images of the LV short axis were acquired for analysis of LV parameters. Left ventricular short-axis late gadolinium enhancement (LGE) images were obtained after 7–10 min of gadopentetate glucosamine injection.

### CMR analysis

LA volume (LAV) is calculated using the two-plane area-length method [24]. LAV includes minimum LAV (LAVmin), maximum LAV (LAVmax), and pre-contraction LAV (LAVpre-ac). LA ejection fraction (LAEF) is calculated with the following equation:

$$\begin{aligned} & {\text{Total LAEF}} = \frac{LAVmax - LAVmin}{{LAVmax}} \times 100\% \\ & {\text{Passive LAEF}} = \frac{LAVmax - LAVpre - ac}{{LAVmax}} \times 100\% \\ & {\text{Active LAEF}} = \frac{LAVpre - ac - LAVmin}{{LAVpre - ac}} \times 100\% \\ \end{aligned}$$

**Fig. 1:**
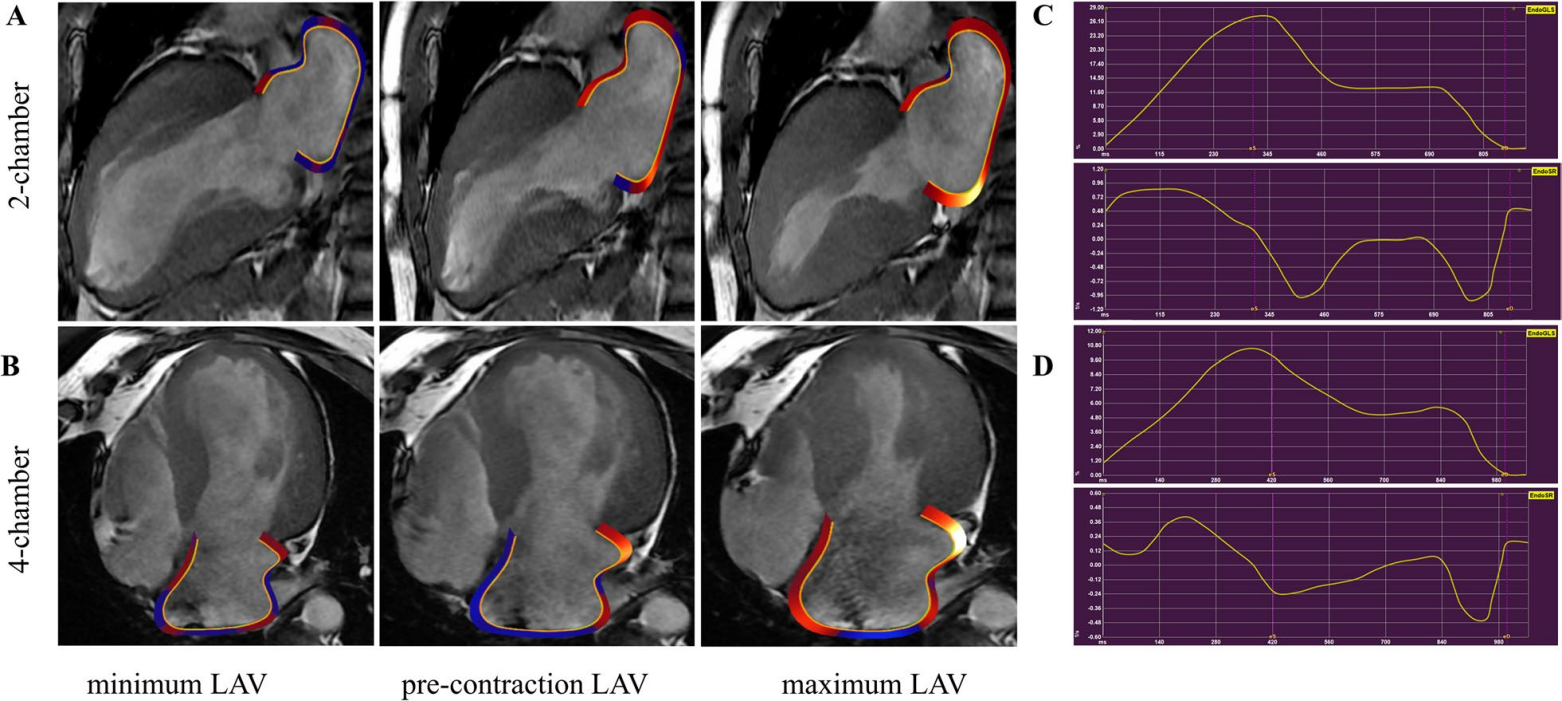
LA tracing in cine cardiac MRI **A** two-chamber and **B** four-chamber views. **C** LA GLS and SR curves for HCM patients without combined adverse clinical events; **D** LA GLS and SR curves for HCM patients with combined adverse clinical events. LA, left atrium; GLS, global longitudinal strain; SR, strain rate

LA strain and strain rate (SR) were acquired using Medis (version 4.0.24, Medis Medical Imaging Systems, Leiden, the Netherlands) post-processing software analysis. The LA endocardial border was manually traced on two- and four-chamber cine images (the presence of pulmonary veins or corresponding segments of the left heart ear were excluded from analysis). A final visual review was performed to ensure accurate tracking of the atrial myocardium. If the automatic boundary tracking was not accurate, the initial contours were manually adjusted and the algorithm was then reapplied (Fig. 1).

LA strain parameters include global longitudinal strain (GLS) and global circumferential strain (GCS). The LA GLS includes reservoir strain (GLS reservoir, corresponding to the atrial reservoir function), conduit strain (GLS conduit, corresponding to the atrial conduit function), and booster strain (GLS booster, the atrial systolic booster pump function). Consequently, three LA GLS SR parameters were derived: SR reservoir (positive strain rate), SR conduit (early negative strain rate), and SR booster (late negative strain rate). LA strain and strain rate were evaluated from 2-chamber and 4-chamber cine images and the average values were calculated.The presence of LGE was visually assessed by two independent observers (DT and ZYL) who were blinded to the clinical data. The post-processing software Medis (version 4.0.24, Medis Medical Imaging Systems, Leiden, the Netherlands) was used for LGE image analysis. The LGE was quantified using the six standard deviations (SD) from the normal myocardium signal intensity and expressed as LGE extent and LGE mass.

### Study endpoint

The primary endpoint was the combined outcome of SCD, new-onset or worsening to hospitalized HF, new-onset AF, thromboembolic events, and fatal ventricular arrhythmias. Direct telephone interviews with patients or family members were conducted by two independent trained clinicians. The follow-up period was defined as the interval between the first CMR clinical evaluation and the February 2022.

### Statistical analysis

Statistical analyses were performed using R (version 4.1.1, R Foundation for Statistical Computing, Vienna, Austria) and SPSS (version 26.0, IBM SPSS Inc, Chicago, IL, USA). The normality of the data was assessed by the Shapiro–Wilk test. For normally and non-normally distributed data, the mean ± standard deviation and median (interquartile range) are presented separately. For normally and non-normally distributed data, independent t-tests and Mann–Whitney tests are used respectively. Categorical variables are expressed as N (%). To compare the proportions of categorical variables, the chi-square test and Fisher's exact test were applied as appropriate. Inter- and intra-observer agreement of the LA strain and SR were assessed by the intra-group correlation coefficient (ICC). Parameters were stratified based on the optimal threshold (cut-off) for predicting composite outcomes for these variables, which was calculated using X-tile software analysis. Univariate Cox proportional risk regression analyses were performed to identify predictors of adverse clinical events. Relative risks were expressed as risk ratio (HR) with 95% confidence intervals (CIs). Parameters at a significance threshold of *p* < 0.05 were included in stepwise multivariate Cox regression analysis to identify potential independent predictive factors. Kaplan–Meier cumulative survival curves without adverse clinical events were constructed for the cut-off points established for parameters of significance in the stepwise regression model. Survival curves were compared using log-rank tests. ROC and decision curve analysis (DCA) were plotted separately at 3-year time points based on time-dependent ROC. A *p* value of 0.05 or lower was considered to indicate a statistically significant difference.

## Results

### Baseline characteristics

**Fig. 2:**
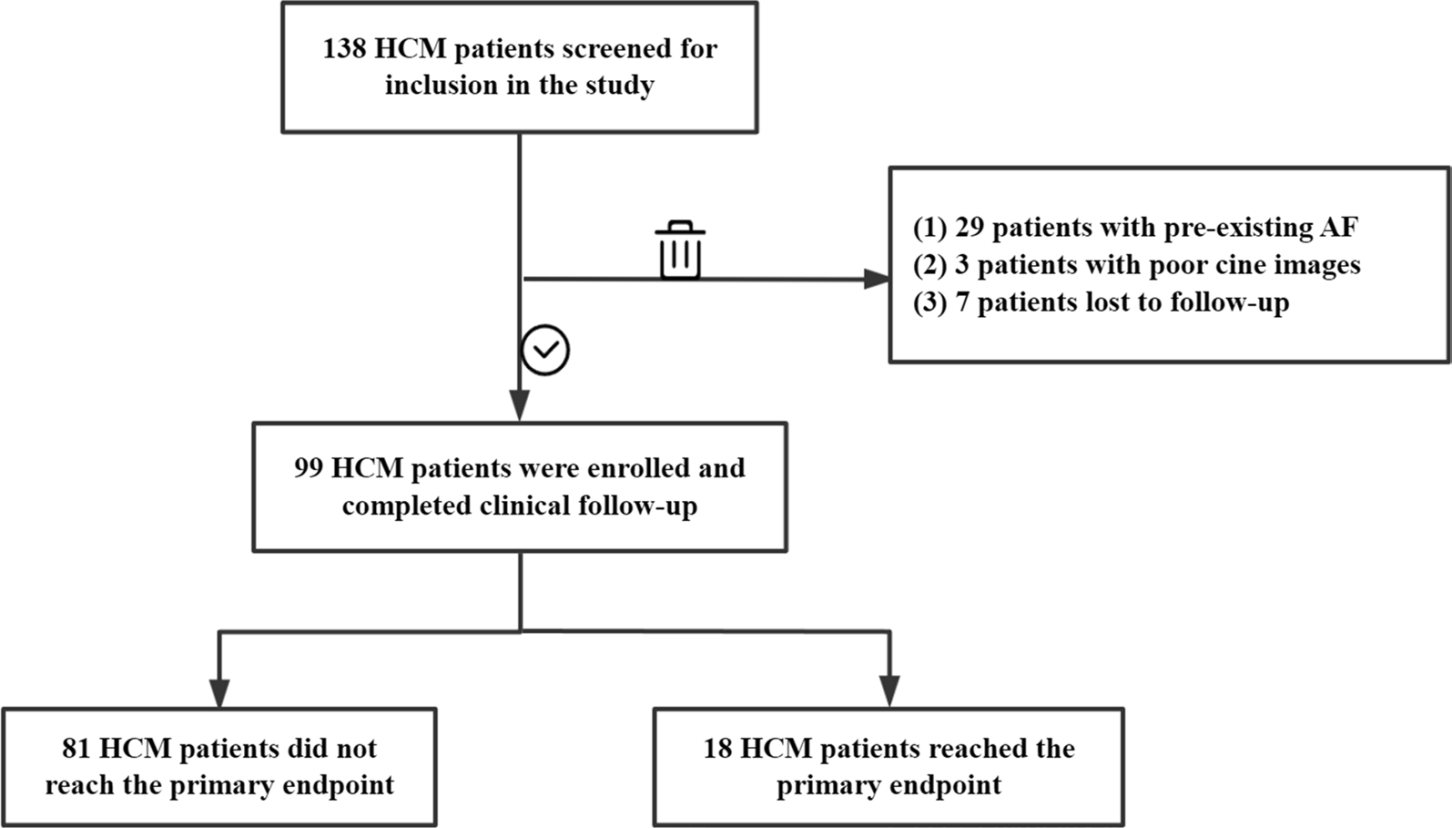
Flow chart for inclusion of HCM patients

A total of 138 patients with HCM who underwent CMR at our institution between May 2012 and September 2021 were retrospectively included. After excluding 29 patients with pre-existing atrial fibrillation, 3 patients with poor images, and 7 patients with no follow-up data, 99 patients with HCM were ultimately included in this study (Fig. 2). A total of 99 patients with HCM (median age 55 years, age range 19–80 years, 71 men (71.1%), BMI 26.40 ± 3.39 kg/m^2^, and heart rate 66 (58,68) beats/min) were collected in this study according to inclusion and exclusion criteria. After a mean follow-up of 37.94 ± 23.69 months, 18 HCM patients reached the primary endpoint, including 10 new-onset AF, 4 new-onset AF with heart failure-related hospitalizations, 1 new-onset AF with acute myocardial infarction, 1 hospitalization for heart failure-related hospitalizations, 1 cardiovascular death and 1 implantable cardioverter-defibrillator (ICD) discharge due to ventricular fibrillation combined with cerebral infarction and heart failure-related hospitalization.

Eighty-nine control subjects (Average age 52.74 ± 10.03years, 67 males [75.28 %], body mass index 26.40 (24.51, 28.40) kg/m^2^, heart rate 68 [64, 75] beats/min) were included. Clinical baseline characteristics and CMR data for HCM patients and control subjects are shown in Tables 1 and 2.

**Table 1:** Basic information on CMR patients: control subjects versus HCM; without adverse clinical events versus with combined outcome

Parameters	Control subjects (n = 89)	HCM population (n = 99)	*p* value	HCM patient without adverse clinical events (n = 81)	HCM patient with adverse clinical events (n = 18)	*p* value
*Baseline and clinical*						
Age at CMR scan, years	52.74 ± 10.03	55 (46, 65)	0.200	51.85 ± 13.13	67 (62, 69)	< 0.001***
Men, n, %	67, 75.28%	71, 71.7%	0.581	61, 75.3%	10, 55.6%	0.092
Family history, n, %	–	31, 31.3%	–	50, 61.7%	12, 66.7%	0.695
Body mass index, kg/m^2^	26.40 (24.51, 28.40)	26.40 ± 3.39	0.821	26.65 ± 3.36	25.28 ± 3.35	0.121
Heart rate, beat/min	68 (64, 75)	66 (58, 68)	< 0.001^***^	67 (59, 71)	60.17 ± 7.52	0.026*
Hypertension, n, %	25, 28.1%	62, 62.6%	< 0.001^***^	50, 61.7%	12, 66.7%	0.695
Systolic blood pressure, mmHg	–	150 (125, 180)	–	150 (123, 180)	159.17 ± 36.81	0.413
Diastolic blood pressure, mmHg	–	90 (77, 110)	–	90 (77, 110)	98.28 ± 26.47	0.420
Diabetes mellitus, n, %	15, 16.9%	17, 17.2%	0.954	14, 17.3%	3, 16.7%	1.000
Smoking history, n, %	44, 49.4%	36, 36.4%	0.070	29, 35.8%	7, 38.9%	0.806
CAD, n, %	–	26, 26.3%	–	22, 27.2%	4, 22.2%	0.721
PE, n, %	–	0, 0%	–	0, 0%	0, 0%	–
CKD, n, %	–	1, 1.01%	–	1, 1.23%	0, 0%	–

Data were expressed as number (percentage), mean ± standard deviation, or median (interquartile range)

CAD, Coronary atherosclerotic heart disease; PE, Pulmonary embolism; CKD, Chronic Kidney Disease

“*”, “**”, and “***” reflect that the difference between groups is statistically significant

“*” represents *p* < 0.05; “**” represents *p* < 0.01; “***” represents *p* < 0.001

**Table 2:** LV and LA parameters and on CMR patients: control subjects versus HCM; without adverse clinical events versus with combined outcome

Parameters	Control subjects (n = 89)	HCM population (n = 99)	*p* value	HCM patient without adverse clinical events (n = 81)	HCM patient with adverse clinical events (n = 18)	*p* value
*LV parameters*						
Maximal LV thickness, mm	–	20.69 (18.67, 24.47)	–	20.53 (18.27, 24.06)	22.47 ± 4.41	0.611
LV EDVI, mL/m^2^	70.70 ± 10.61	77.77 (69.30, 87.04)	< 0.001***	78.67 (68.75, 88.35)	74.03 (68.95, 84.95)	0.717
LV ESVI, mL/m^2^	26.54 ± 5.72	30.23 (25.68, 38.08)	< 0.001***	29.73 (25.66, 36.77)	34.59 (25.75, 45.93)	0.292
LV EF, %	62.44 ± 6.05	59.88 (52.95, 65.74)	0.008**	60.55 (53.28, 65.79)	53.51 ± 13.26	0.147
LV SV, ml	84.06 ± 17.13	86.14 (72.85, 100.20)	0.310	87.92 (74.48, 105.69)	77.44 ± 20.81	0.030**
LV CO, L/min	5.57 (5.06, 6.62)	5.47 (4.47, 6.64)	0.337	5.69 (4.65, 6.81)	4.64 ± 1.24	0.005**
LV M, g	97.94 ± 19.22	165.25 (125.22,222.86)	< 0.001***	161.79 (126.41, 220.79)	187.18 ± 78.45	0.877
*LA volume and function*						
LA diameter, mm	39.82 ± 4.63	49.77 ± 6.87	< 0.001***	49.34 ± 6.47	52.01 ± 8.41	0.218
LAV max, ml	77.02 ± 19.62	98.99 ± 27.35	< 0.001***	95.69 ± 26.85	113.84 ± 25.19	0.010*
LAV pre-ac, ml	55.71 ± 14.91	81.18 ± 25.87	< 0.001***	76.87 ± 24.59	92.05 (84.60, 112.41)	< 0.001***
LAV min, ml	33.23 ± 10.53	55.86 ± 21.71	< 0.001***	51.62 ± 19.33	74.90 ± 22.14	< 0.001***
Total LAEF, %	57.16 ± 5.89	45.61 (39.06, 51.80)	< 0.001***	46.99 ± 9.41	34.67 ± 9.29	< 0.001***
Passive LAEF, %	27.15 ± 5.84	18.50 ± 8.23	< 0.001***	20.07 ± 7.86	11.45 ± 5.94	< 0.001***
Active LAEF, %	40.94 ± 5.38	34.34 (27.31, 38.29)	< 0.001***	34.97 (29.08, 39.07)	26.17 ± 8.48	< 0.001***
*LA strain*						
GLS reservoir, %	32.10 (27.06, 37.42)	21.68 ± 7.19	< 0.001***	23.129 ± 6.84	15.19 ± 4.83	< 0.001***
GLS conduit, %	16.00 ± 4.35	8.95 (6.86, 12.84)	< 0.001***	10.16 (7.32, 14.12)	6.04 ± 2.57	< 0.001***
GLS booster, %	16.26 ± 3.20	11.57 ± 3.90	< 0.001***	12.11 ± 3.81	9.15 ± 3.43	0.003**
GCS, %	34.57 ± 8.47	24.83 ± 10.13	< 0.001***	26.67 ± 9.49	16.55 ± 8.90	< 0.001***
*LA strain rate*						
SR reservoir, s^−1^	1.12 ± 0.24	0.79 ± 0.25	< 0.001***	0.83 ± 0.24	0.61 ± 0.21	0.001***
SR conduit, s^−1^	− 0.978 (− 1.28, − 0.81)	− 0.59 (− 0.81, − 0.44)	< 0.001***	− 0.64 (− 0.85, − 0.50)	− 0.44 ± 0.20	< 0.001***
SR booster, s^−1^	− 1.51 ± 0.33	− 1.00 ± 0.34	< 0.001***	− 1.06 ± 0.32	− 0.72 ± 0.27	< 0.001***

Data were expressed as number (percentage), mean ± standard deviation, or median [interquartile range]

LV, left ventricular; EDVI, end-diastolic volume index; ESVI, end-systolic volume index; EF, ejection fraction; SV, stroke volume; CO, cardiac output; M, myocardial mass; LA, left atrium; LAV, left atrial volume; LAEF, left atrial ejection fraction; GLS, global longitudinal strain; GCS, global circumferential strain; SR, strain rate

“*”, “**”, and “***” reflect that the difference between groups is statistically significant

“*” represents *p* < 0.05; “**” represents *p* < 0.01; “***” represents *p* < 0.001

### Outcomes

Compared to control subjects, patients with HCM had larger LA diameter, larger LV end-diastolic volume index (EDVI), larger LV end-systolic volume index (ESVI), larger LV myocardial mass (M), larger LAV, lower LV EF, and lower LAEF (all *p* < 0.05). Ninety-nine patients with HCM included 18 cases with LVEF < 50%. The values of GLS (reservoir, conduit, booster), SR (reservoir, conduit, booster), and GCS in HCM patients were significantly lower than those of control subjects (all *p* < 0.001).

Compared to those without adverse clinical events, patients with adverse clinical events were older and had greater LAV, lower BSA, lower heart rate, lower LV stroke volume (SV), lower LV cardiac output (CO), lower LAEF, lower GLS (reservoir, conduit, and booster), lower SR (reservoir, conduit, and booster) and lower GCS (all *p* < 0.05).

### Association of LA strains with adverse clinical events

To assess the determinants of adverse clinical events in patients with HCM, univariate Cox regression analysis, and stepwise multivariate proportional hazard analysis were performed (see Table 3). In univariable Cox regression analysis, the following variables were found to be significant predictors of adverse clinical events: age at CMR scan, body mass index, heart rate, LV ESVI, LV EF, LV SV, LV CO, LA diameter, LAV, (total, passive, active) LAEF, LA GLS (reservoir, conduit, booster), LA SR (reservoir, conduit, booster), and LA GCS (all *p* < 0.05).

**Table 3:** Univariate Cox and stepwise multivariate Cox regression analysis of risk factors for adverse clinical events in patients with HCM

Parameters	Univariate analysis			Stepwise regression analysis		
	HR	95% CI	*p* value	HR	95% CI	*p* value
*Baseline and clinical*						
Age at CMR scan (continuous)	7.716	(2.740, 21.723)	< 0.001***			
Age at CMR scan (threshold)	1.096	(1.042, 1.153)	< 0.001***			
Men, n, %	0.530	(0.530,0.209)	0.182			
Family history, n, %	0.844	(0.300, 2.371)	0.748			
Body mass index, kg/m^2^(continuous)	0.882	(0.768, 1.011)	0.072			
Body mass index, kg/m^2^(threshold)	2.745	(1.081, 6.971)	0.034*			
Heart rate, beat/min (continuous)	0.930	(0.875, 0.988)	0.020*			
Heart rate, beat/min (threshold)	3.986	(1.442, 11.017)	0.008**	4.137	(0.945, 18.112)	0.059
Hypertension, n, %	1.380	(0.518, 3.679)	0.520			
Diabetes mellitus, n, %	0.898	(0.260, 3.105)	0.865			
Smoking history n, %	1.194	(0.462, 3.083)	0.714			
CAD, n, %	0.957	(0.313, 2.923)	0.938			
*LV parameters*						
Maximal LV thickness (continuous)	1.017	(0.950, 1.088)	0.624			
Maximal LV thickness (threshold)	2.304	(0.912, 5.816)	0.077			
LVEDVI (continuous)	0.999	(0.979, 1.019)	0.918			
LVEDVI (threshold)	1.397	(0.554, 3.524)	0.478			
LVESVI (continuous)	1.006	(0.989, 1.024)	0.466			
LVESVI (threshold)	3.488	(1.335, 9.114)	0.011*			
LVEF (continuous)	0.980	(0.953, 1.008)	0.167			
LVEF (threshold)	2.788	(1.093, 7.110)	0.032*			
LVSV (continuous)	0.984	(0.967, 1.000)	0.047*			
LVSV (threshold)	3.102	(1.161, 8.283)	0.024*	7.654	(1.642, 35.682)	0.010**
LVCO (continuous)	0.720	(0.566,0.916)	0.008**			
LVCO (threshold)	3.677	(1.424, 9.495)	0.007**			
LVM (continuous)	1.001	(0.995, 1.007)	0.805			
LVM (threshold)	1.394	(0.540, 3.600)	0.493			
LVMI (continuous)	1.005	(0.994, 1.016)	0.387			
LVMI (threshold)	2.132	(0.841, 5.406)	0.111			
*LA volume and function*						
LA diameter (continuous)	1.046	(0.976, 1.120)	0.204			
LA diameter (threshold)	4.046	(1.558, 10.506)	0.004**	4.981	(1.223, 20.297)	0.025**
LAV max, ml (continuous)	1.021	(1.004, 1.038)	0.014*			
LAV max, ml (threshold)	4.680	(1.069, 20.493)	0.041*			
LAV pre-ac (continuous)	1.032	(1.014, 1.051)	< 0.001***			
LAV pre-ac (threshold)	6.248	(2.050, 19.042)	0.001**	4.175	(1.252,13.926)	0.020**
LAV min (continuous)	1.038	(1.020, 1.055)	< 0.001***			
LAV min (threshold)	5.299	(1.879, 14.939)	0.002**			
Total LAEF (continuous)	0.897	(0.857,0.938)	< 0.001***			
Total LAEF (threshold)	6.600	(2.570, 16.950)	< 0.001***			
Passive LAEF (continuous)	0.844	(0.779,0.915)	< 0.001***			
Passive LAEF (threshold)	10.198	(3.957, 26.280)	< 0.001***	8.808	(2.876, 26.980)	< 0.001***
Active LAEF (continuous)	0.919	(0.877,0.964)	0.001**			
Active LAEF (threshold)	5.666	(2.099, 15.295)	0.001**			
*LA strain*						
GLS reservoir (continuous)	0.828	(0.763,0.899)	< 0.001***			
GLS reservoir (threshold)	9.155	(3.295, 25.442)	< 0.001***			
GLS conduit (continuous)	0.685	(0.570,0.823)	< 0.001***			
GLS conduit (threshold)	5.602	(1.992, 15.750)	0.001**			
GLS booster (continuous)	0.819	(0.726, 0.925)	0.001**			
GLS booster (threshold)	8.132	(2.911, 22.712)	< 0.001***	8.936	(1.951, 40.933)	0.005**
GCS (continuous)	0.881	(0.825,0.940)	< 0.001***			
GCS (threshold)	9.728	(3.797, 24.925)	< 0.001***			
LA strain rate						
SR reservoir (continuous)	0.010	(0.001, 0.108)	< 0.001***			
SR reservoir (threshold)	4.147	(1.620, 10.615)	0.003**			
SR conduit (continuous)	0.005	(0,0.071)	< 0.001***			
SR conduit (threshold)	6.149	(2.427, 15.582)	< 0.001***			
SR booster (continuous)	0.053	(0.013,0.220)	< 0.001***			
SR booster (threshold)	7.062	(2.620, 19.031)	< 0.001***			

HR, hazard ratio; CAD, Coronary atherosclerotic heart disease; LV, left ventricular; EDVI, end-diastolic volume index; ESVI, end-systolic volume index; EF, ejection fraction; SV, stroke volume; CO, cardiac output; M, myocardial mass; MI, myocardial mass index; LA, left atrium; LAV, left atrial volume; LAEF, left atrial ejection fraction; GLS, global longitudinalstrain; GCS, global circumferential strain; SR, strain rate

“*”, “**”, and “***” reflect that the difference between groups is statistically significant

“*” represents *p* < 0.05; “**” represents *p* < 0.01; “***” represents *p* < 0.001

A stepwise regression model was constructed by a forward conditional algorithm selecting variables from the baseline and CMR variables and contained six independent predictors: heart rate (HR = 4.137 [95% CI 0.945, 18.112], *p* = 0.059), LV SV (HR = 7.654 [95% CI 1.642, 35.682], *p* = 0.010), LA diameter (HR = 4.981 [95% CI 1.223, 20.297], *p* = 0.025), LAV pre-ac (HR = 4.175 [95% CI 1.252,13.926], *p* = 0.020), passive LAEF (HR = 8.808 [95% CI 2.876, 26.980], *p* < 0.001), and LA GLS booster (HR = 8.936 [95% CI 1.951, 40.933], *p* = 0.005). LA GLS booster was a stronger predictor than the other factors.

#### Kaplan–Meier curves

**Fig. 3:**
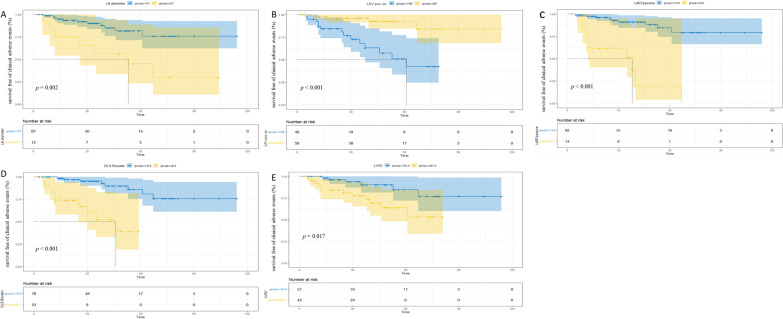
Kaplan–Meier curves represent survival of HCM patients who were free of adverse clinical events. Participants with **A** LA diameter of more than or equal to 57 mm, **B** LAV pre-ac of more than or equal to 85 ml, **C** passive LAEF of less than or equal to 9.6%, **D** LA GLS booster of less than or equal to 8.9%, and **E** LV SV of less than or equal to 83.4 ml displayed significantly higher risk of adverse clinical events. LA, left atrium; LAV, left atrial volume; pre-ac, pre-contraction; LAEF, left atrial ejection fraction; GLS, global longitudinal strain; LV, left ventricular; SV, stroke volume

Survival graphs displaying survival free from adverse clinical events were produced for the variables that reached significance in the stepwise regression model (Fig. 3). The best cut-off values for LA diameter, LAV pre-ac, passive LAEF, GLS booster, and LV SV predicted combined results were 57 mm, 85 ml, 9.6%, 8.9%, and 83.4 ml, respectively. Composite event-free survival was significantly lower with LA diameter ≥ 57 mm (*p* = 0.002), LAV pre-ac ≥ 85 ml (*p* < 0.001), passive LAEF ≤ 9.6% (*p* < 0.001), GLS booster ≤ 8.9% (*p* < 0.001), and LV SV ≤ 83.4 ml (*p* = 0.017) in HCM patients.

### Predictive value and risk probability of adverse clinical events predicted by LA strain at the 3-year time point

**Fig. 4:**
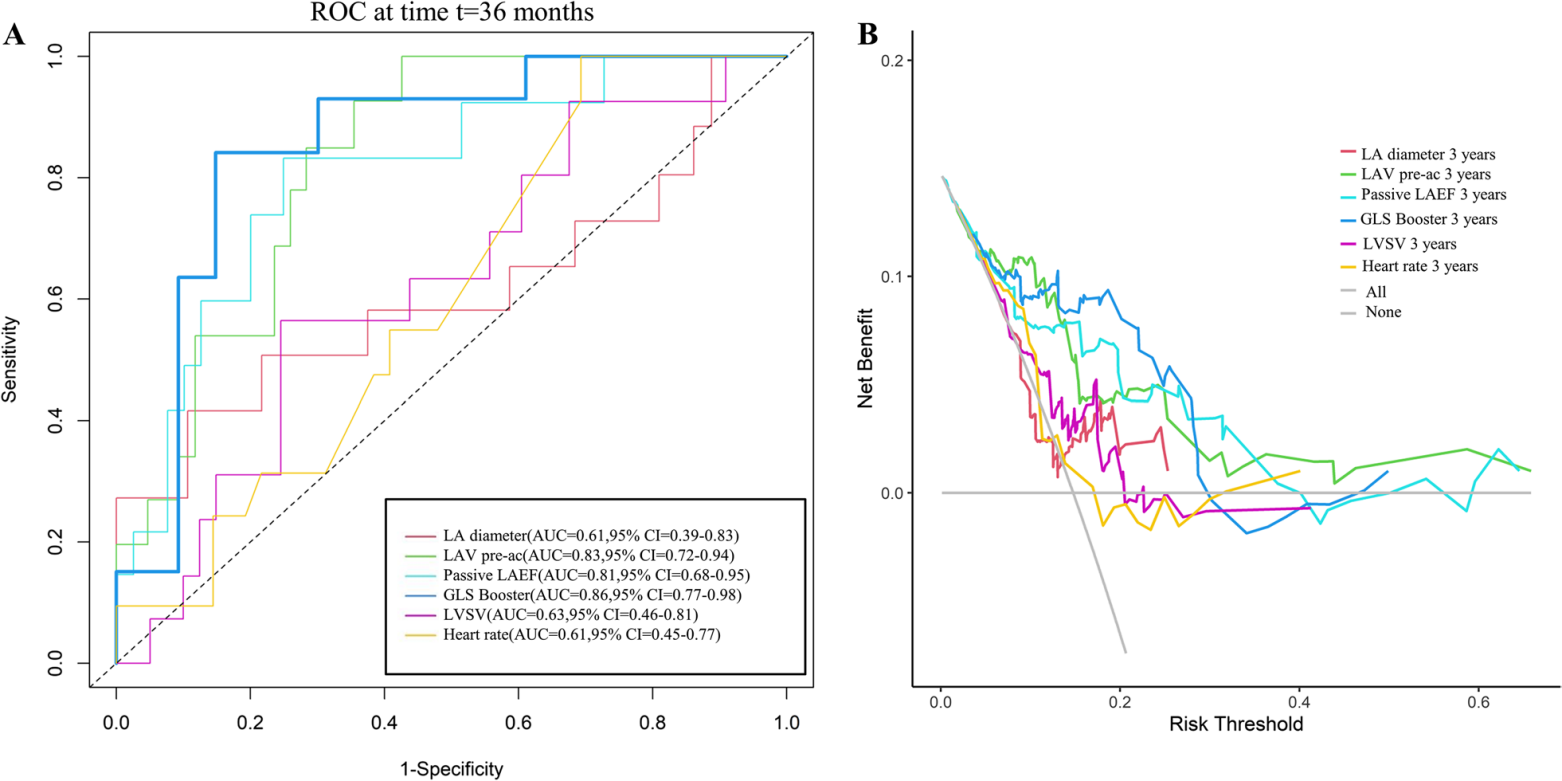
Predictive performance and clinical utility of the parameters in the stepwise regression model. **A** Receiver operating characteristic curves of LA diameter, LAV pre-ac, LAEF passive, LA GLS booster, LV SV, and heart rate for prediction of adverse clinical events at 3 years; **B** decision curve analysis of LA diameter, LAV pre-ac, LAEF passive, LV SV, LA GLS booster and heart rate at 3 years to predict adverse clinical events in HCM patients. The y-axis represents the net benefit. The x-axis represents the threshold probability, which means that the expected benefit of treatment is equivalent to the expected benefit of non-treatment. The numbers in parentheses are the areas under the receiver operating characteristic curves (AUCs) and 95% confidence intervals (CIs). LA, left atrium; LAV, left atrial volume; pre-ac, pre-contraction; LAEF, left atrial ejection fraction; GLS, global longitudinal strain; LV, left ventricular; SV, stroke volume

**Fig. 5:**
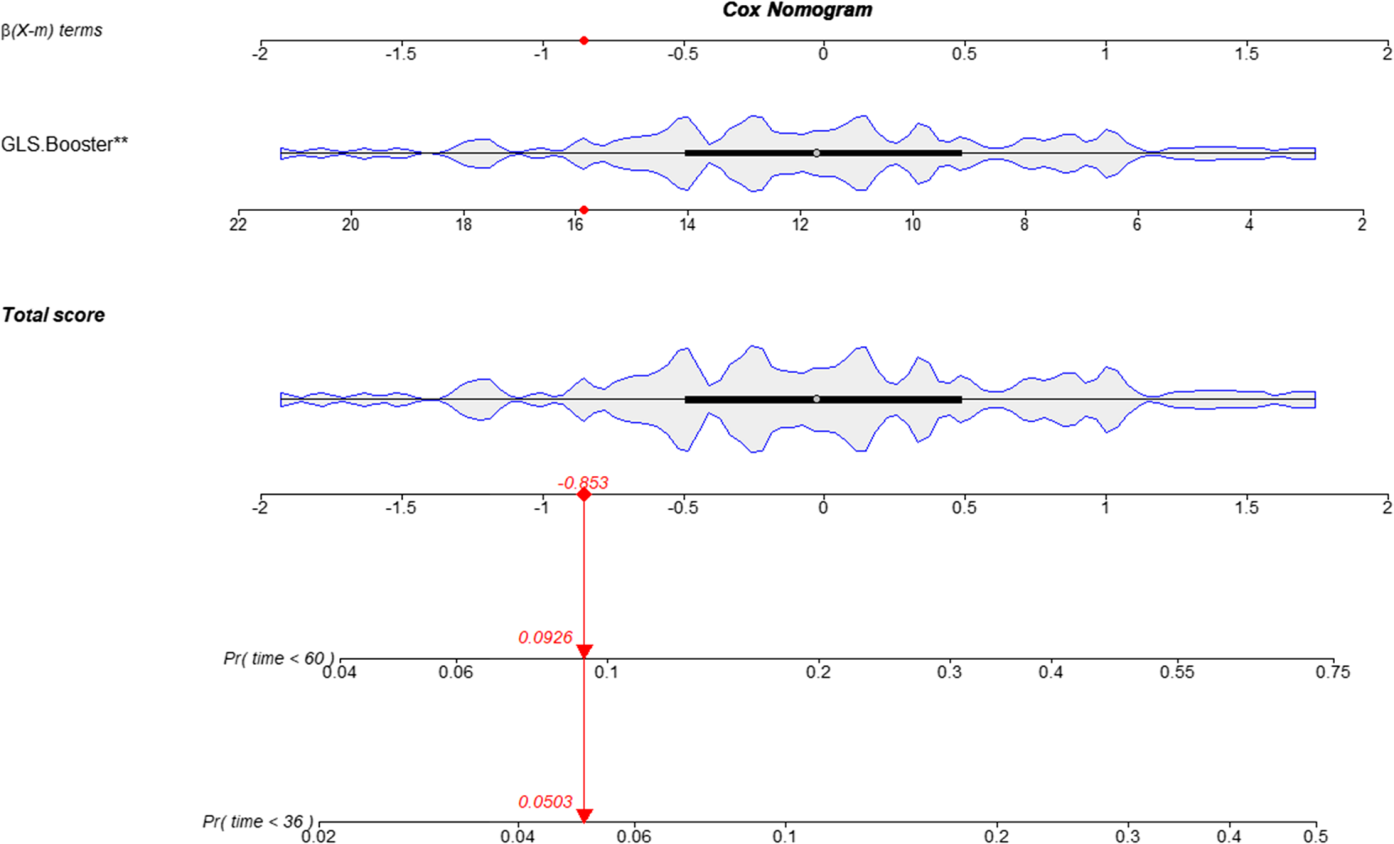
LA GLS booster Cox nomogram. LA, left atrium; GLS, global longitudinal strain; “^**^” represents *p* < 0.01

Figure 4A shows the ROC curves based on LV SV, LA diameter, LAV pre-ac, passive LAEF, and LA GLS booster for predicting adverse clinical events in HCM patients at 3 years. The area under the ROC curve (AUC) for LA GLS booster (AUC = 0.86 [95%CI 0.77–0.98]) is greater than the other indicators. The DCA curve allows inference of the probability of risk of adverse clinical events in patients with HCM at 3 years (Fig. 4B). In addition, a GLS booster Cox nomogram has been constructed to facilitate clinical decision analysis (Fig. 5).

### LGE subgroup analysis

For this study, a total of 83 patients underwent LGE scans, and three of them were excluded from the subgroup analysis because of the poor quality of LGE images. Finally, 80 patients with HCM were analyzed in the LGE subgroup, including 54 cases LGE positive (LGE +) and 26 cases LGE negative (LGE-). Eighteen adverse clinical events occurred in 80 HCM cases. There were 54 patients with LGE + HCM with LGE extent of 4.41 (2.91, 10.24) % and LGE mass of 6.15 (3.50, 15.28) g. Fifteen cases in the 54 LGE + HCM patients reached the primary endpoint."

**Fig. 6:**
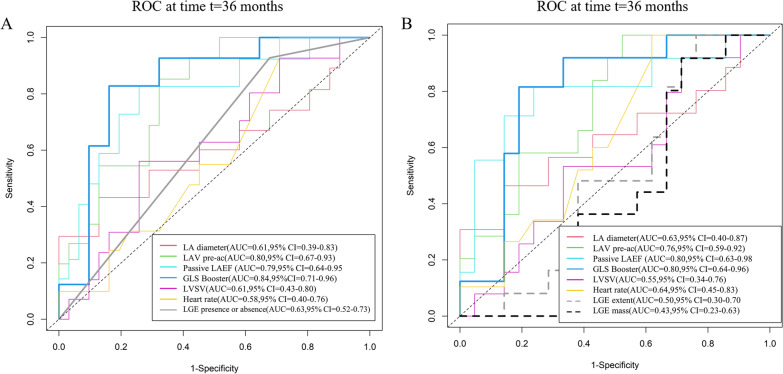
Subgroup receiver operating characteristic curves at three years. **A** ROC curves for LA diameter, LAV pre-ac, Passive LAEF, LA GLS booster, LV SV, heart rate, and LGE presence or absence predict adverse clinical events at three years in a subgroup of 80 HCM patients with LGE scans. **B** Receiver operating characteristic curves for LA diameter, LAV pre-ac, Passive LAEF, LA GLS booster, LV SV, heart rate, LGE extent, and LGE mass predict adverse clinical events at three years in a subgroup of 54 LGE + HCM patients. LA, left atrium; LAV, left atrial volume; pre-ac, pre-contraction; LAEF, left atrial ejection fraction; GLS, global longitudinal strain; LV, left ventricular; SV, stroke volume

In the subgroup of 80 HCM patients with LGE scans (Fig. 6A), the AUC for LGE presence or absence predicting adverse clinical events at the 3-year time point was 0.63 (95% CI [0.52–0.73]), while LA GLS booster (AUC = 0.84,95% CI [0.71–0.96]) still maintained optimal diagnostic efficacy. In the subgroup of 54 LGE + HCM patients (Fig. 6B), the performance of LGE extent (AUC = 0.50 (95% CI [0.30–0.70])) and LGE mass (AUC = 0.43 (95% CI [0.23–0.63]) in predicting adverse clinical events in HCM at the 3-year time point was lower than each parameter in the stepwise regression model, and LA GLS booster's predictive performance (AUC = 0.80, 95% CI [0.63–0.98]) remained stable.

### Reproducibility of LA strain measurements

Fifteen HCM patients and twenty-five control subjects were randomly selected from the study group to assess intra- and inter-observer agreement (Table 4). The results showed intra-group ICCs of 0.850–0.976 and inter-group ICCs of 0.891–0.972 for LA strain and SR (all ICCs ≥ 0.85).

**Table 4:** Agreement of LA strain and strain rate analysis (N = 40)

Parameters	Intra-group		Inter-group	
	ICC	CI	ICC	CI
*LA strain*				
GLS reservoir, %	0.969	0.942–0.983	0.941	0.892–0.969
GLS conduit, %	0.957	0.920–0.977	0.952	0.911–0.974
GLS booster, %	0.949	0.905–0.972	0.891	0.803–0.941
GCS, %	0.850	0.736–0.918	0.911	0.837–0.952
*LA strain rate*				
SR reservoir, s^−1^	0.906	0.829–0.949	0.859	0.751–0.923
SR conduit, s^−1^	0.976	0.955–0.987	0.972	0.948–0.985
SR booster, s^−1^	0.939	0.887–0.967	0.895	0.811–0.943

LA, left atrium; LAV, left atrial volume; LAEF, left atrial ejection fraction; GLS, global longitudinal strain; GCS, global circumferential strain; SR, strain rate

### Three studies of CMR LA strain predict adverse clinical events in HCM patients

A summary of studies using CMR LA strain to predict adverse clinical events in patients with HCM is shown in Table 5.

**Table 5:** Summary of patient demographics, study characteristics, and study findings for the three CMR studies

Author, year	HCM, n	Control, n/matched by	LA strain measurement software	Measurement method	Views of measured LA strain	Follow-up, months	Key findings
Hinojar et al. [13], 2018	75	75/age and sex	Cvi 42	Standard CMR-FT	2-, 3- and 4-chamber views	39.6	LA longitudinal strain by CMR-FT may become a novel potential predictor of poor cardiac outcomes
Yang et al. [14], 2021	359	100/age	Medis2.0, Qstrain	A fast LA long-axis strain method	2-and 4-chamber views	40.9	Fast LA GLS reservoir and conduit emerged as independent predictors of the composite adverse events
Zhou et al. [23], 2022	60	60/sex	Medis3.1, QStrain	Standard CMR-FT	2-, 3- and 4-chamber views	81.6	Impaired LA GLS reservoir and booster were associated with clinical outcomes in patients at the early stage of hypertension and HCM

HCM, hypertrophic cardiomyopathy; CMR, cardiac magnetic resonance; NR, no reported; CMR-FT, cardiac magnetic resonance feature tracking LA, left atrium; GLS, global longitudinal strain

### Discussion

In our study, the predictive utility of LA strain metrics was assessed in a CMR cohort of HCM participants with a mean follow-up of 37.94 ± 23.69 months, with the following results. First, LA strain and SR were significantly impaired in HCM participants compared with age-, sex-, and BMI-matched controls. Second, stepwise multivariate proportional hazard analysis showed that LV SV, LA diameter, LAV pre-ac, passive LAEF, and LA GLS booster were independent predictors of adverse clinical events in HCM participants. Compared to other parameters, patients with LA GLS booster dysfunction have a higher risk of adverse clinical events. Third, HCM participants with LA GLS booster ≤ 8.9% had a significantly higher long-term risk of adverse clinical events than those with GLS booster strain > 8.9% according to Kaplan–Meier survival analysis. Finally, LA GLS booster provided greater predictive value in predicting adverse clinical events in HCM participants at the 3-year time point compared to traditional CMR indexes.

Studies have shown that in addition to LA size and LAV, measuring LA strain can better reflect LA fibrosis, remodeling, and its underlying pathophysiological changes [10, 11, 25]. LA strain provides an alternative method of measuring atrial mechanics and has been found to potentially provide greater insight into the risk of arrhythmias, thromboembolic events, and other adverse outcomes in the general population and patients with cardiovascular disease [26–29]. With new evidence supporting the emergence of deformation imaging, the European Association of Cardiovascular Imaging (EACVI)/ American Society of Echocardiography (ASE)/Industry Task Force reached a consensus to standardize left atrial, right ventricular, and right atrial deformation imaging [30]. In short, the consensus recommends the use of the standard four-chamber and two-chamber views method for determining LA strain. These are the views chosen by most CMR studies to measure LA strain [17, 31].Therefore, our study used LA strain acquired from CMR standard two-chamber and four-chamber cine images to predict adverse clinical events in patients with HCM.

This study found that LA GLS reservoir, conduit, and booster were all impaired in HCM patients compared to control subjects, which is consistent with previous findings [14, 32, 50]. There are two possible mechanisms that explain why strain was significantly lower in the HCM group than in the control group. First, patients with HCM may lead to LA fibrosis and subsequent reduced LA compliance, resulting in impaired LA strain [33]. Second, HCM patients may experience progressive LA remodeling and dysfunction [34]. However, some studies have shown significant differences between HCM patients and healthy control subjects for LA GLS reservoir and conduit, but not LA GLS booster [18, 23, 35]. One possible explanation for this difference is that the preserved LA pumping function is a compensatory mechanism to maintain the stroke volume and left ventricular filling in mild diastolic insufficiency, and its deterioration reflects LA compliance in the "decompensated" phase [35, 36].

Similarly, previous studies have shown that patients with HCM with adverse clinical events have significantly lower LA GLS reservoir, conduit, and booster than patients without adverse clinical events [12, 22], and the same result was obtained in our study. However, Vasquez et al. [37] and Yang et al. [14] found that there was no statistically significant difference in LA GLS booster in HCM patients who developed adverse clinical events compared to those who did not. Vasquez et al. [37] included some HCM patients with paroxysmal atrial fibrillation (PAF) who had a worse course and more severe complications than those without PAF [38], which may lead to bias when further grouping occurs. Meanwhile, Yang et al. [14] used a fast LA long-axis strain method for quantifying long LA deformation. The differences in the results of studies may be related to the cases enrolled and how they were measured.

In this study, univariate Cox regression analyses showed that LA GLS reservoir, conduit, and booster were all significant predictors of adverse clinical events in patients with HCM. Multivariate Cox regression analysis evidence suggests that LA GLS booster is an independent predictor, rather than LA GLS reservoir and conduit, and can be used as an imaging marker to predict adverse clinical events in HCM patients. Previous studies have shown that LA booster pump function is more important in ventricular disease (LV dysfunction, myocardial infarction, hypertensive heart disease, and non-ischaemic cardiomyopathy)[39–42], where it preserves cardiac output [39] and helps to control pulmonary capillary wedge pressure [40]. Fujimoto et al. [43] a STE study of 76 patients with HCM showed that LA GLS booster was independently associated with cardiac events such as HF-related hospitalization and AF during a follow-up of 2.6 ± 1.7 years, which is consistent with our findings. Nevertheless, LA strain predicts adverse clinical events in patients with HCM with not very consistent results. Zhou et al. [23] retrospectively studied CMR images in 60 patients with HCM and found that LA GLS reservoir and booster were associated with the adverse outcomes of sudden cardiac deaths, new-onset or worsening of HF to hospitalizations, and paroxysmal or persistent AF during a mean follow-up of 6.8 ± 2.1 years, which is partially consistent with our results. However, Yang et al. [14] conducted a CMR-FT study of HCM patients using a fast LA-LAS method to assess LA strain and found that LA GLS reservoir and conduit were associated with cardiovascular deaths, SCD aborted by appropriate ICD discharge, resuscitations after syncope, and hospital admissions related to HF. Vasquez et al. [37] a STE study of 94 patients with HCM showed that low LA GLS reservoir and conduit were found to be associated with adverse outcomes of HF, stroke, and death at 5.8 ± 3.3 years of follow-up. These two studies did not find a significant correlation between LA GLS booster and adverse clinical outcomes. First, these differences between studies may be attributed to the heterogeneity of the underlying patient data, differences in the CMR views selected, and differences in measurement methods. Second, LA pump function is based on LA intrinsic systolic and LV end-diastolic compliance and LV pressure [36]. HCM manifests as ventricular hypertrophy, excessive myocardial contraction, myocardial fibrosis, and reduced compliance[44], resulting in increased LV pressure and increased LA afterload (pressure). Studies have shown that in patients with heart failure with preserved ejection fraction, LA pump function can directly decompress high left atrial pressure and associated pulmonary congestion and improve systemic blood flow [45]. In our study, LVEF was significantly lower in the adverse clinical events group, which may lead to a progression of LA pump function toward "decompensation".

AF is the most common arrhythmic event in patients with HCM, and AF is associated with low cardiovascular mortality concerning heart failure, arrhythmic sudden death, or thromboembolism [46, 47]. During the follow-up of this study, the majority of adverse clinical events in HCM patients were associated with the occurrence of AF. Earlier studies found LA diameter, LAV, to be a predictor of new-onset AF in patients with HCM [6, 8]. Raman et al. [22] indicated that the diagnostic performance of LA GLS reservoir (AUC = 0.78) and booster (AUC = 0.71) in predicting new-onset atrial fibrillation in HCM patients at the 3-year time point were superior or equal to that of the conventional LA parameter (AUC = 0.60–0.71). Similarly, our study identified independent predictors of future adverse clinical event occurrence in HCM, specifically LA GLS Booster, whose diagnostic performance at the 3-year time point was superior to conventional LA parameters.

A CMR study by Leng et al. [19] assessing LA strain of patients with ST-Segment Elevation Myocardial Infarction (STEMI) showed that in the ROC curve predicting major adverse cardiac events at 3 years, the AUCs of LA GLS reservoir (AUC = 0.75) and conduit (AUC = 0.75) were greater than that of other conventional metrics. In addition, the 2020 AHA/ACC guideline [48] states that patients with HCM should undergo risk assessment based on CMR every 3–5 years. Therefore, ROC curves and DCA curves were constructed for each parameter at the 3-year time point in our study. The AUC of LA GLS booster at the 3-year time point for predicting adverse clinical events in patients with HCM was 0.86. Subsequently, LA GLS booster Cox nomogram was created, which can be used more intuitively and easily to guide clinical decision-making. In addition, the presence and extent of LGE have also been associated with an increased risk of adverse events in HCM. Extensive LGE may thus be considered a new risk marker that could help identify high-risk patients [49]. In our study, we performed a subgroup analysis because some HCM did not have LGE images. This study showed that the AUC of LA GLS booster to predict adverse clinical events at three years remained stable, both in the subgroup of 80 patients with LGE scans and in 54 LGE + HCM patients.

In the contemporary series of patients with HCM from adolescence to adulthood, the annual disease-related mortality rate is estimated to be 0.5% [4]. Therefore, it is necessary to further investigate the risk predictors and models of adverse clinical events in HCM patients to provide clinical guidance. In this study, impaired LA strain was associated with an increased risk of adverse clinical events in HCM patients, demonstrating the utility of LA strain in preventing adverse clinical events. The statistical selection algorithm identified LA GLS booster as the strongest predictor of adverse clinical events in HCM patients, and these suggest that LA strain provides additional predictive information in HCM beyond the basic clinical information, conventional CMR parameters. In addition, a score is assigned to the value of LA GLS booster of the acquired HCM patients according to the degree of contribution of LA GLS booster to the outcome variables in the Cox nomogram; finally, this score is used to predict the probability of the individual's risk of an outcome event at 3 or 5 years. In clinical practice, physicians can be able to quickly query the future risk probability of a patient based on the LA GLS booster values, which facilitates clinical interventions for the patient.

Some studies have shown good intergroup reproducibility and feasibility of LA strain and strain rate both in the normal population and in patients with HCM [18, 50]. This is consistent with our findings, indicating good stability of LA strain and strain rate in this study.

### Limitations

Our study also has some limitations. First, this was a single-center study and our sample size was inadequate, with only 99 HCM patients and 89 controls included in the study, which is far from being considered a large enough sample set. Second, due to the short follow-up period and the small number of SCD events, the risk of SCD could not be examined in this study. Third, this study lacks data on the association of CMR with STE. Fourth, no external validation of thresholds for LA parameters were performed, so the selected thresholds need to be interpreted with caution when extrapolating to all HCM patients.

## Conclusions

In conclusion, LA GLS booster is associated with adverse clinical outcomes in patients with HCM and provides important predictive information.

## Data Availability

The datasets used and/or analysed during the current study are available from the corresponding author on reasonable request.

## Abbreviations

HCM: Hypertrophic cardiomyopathy
AF: Atrial fibrillation
CMR: Cardiac magnetic resonance
LA: Left atrial/ atrium
SCD: Sudden cardiac death
HF: Heart failure
STE: Speckle tracking echocardiography
CMR-FT: Cardiac magnetic resonance feature tracking
ROC: Receiver operating characteristic
LV: Left ventricular
LGE: Late gadolinium enhancement
LAV: Left atrial volume
LAVmin: Minimum LAV
LAVpre-ac: Pre-contraction LAV
LAEF: LA ejection fraction
SR: Strain rate
GLS: Global longitudinal strain
GCS: Global circumferential strain
ICC: Intra-group correlation coefficient
HR: Risk ratio
CIs: Confidence intervals
EDVI: End-diastolic volume index
ESVI: End-systolic volume index
M: Myocardial mass
SV: Stroke volume
CO: Cardiac output
AUC: The area under the ROC curve
EACVI: European Association of Cardiovascular Imaging: American Society of Echocardiography
ASE: American Society of Echocardiography
PAF: Paroxysmal atrial fibrillation
